# Clinical evidence based review and recommendations of aerosol generating medical procedures in otolaryngology – head and neck surgery during the COVID-19 pandemic

**DOI:** 10.1186/s40463-020-00425-6

**Published:** 2020-05-06

**Authors:** Andrew Thamboo, Jane Lea, Doron D. Sommer, Leigh Sowerby, Arman Abdalkhani, Christopher Diamond, Jennifer Ham, Austin Heffernan, M. Cai Long, Jobanjit Phulka, Yu Qi Wu, Phillip Yeung, Marc Lammers

**Affiliations:** 1 Division of Otolaryngology Head & Neck Surgery, Department of Surgery, University of British Columbia, Vancouver, BC Canada; 2 Division of Otolaryngology Head & Neck Surgery, Department of Surgery, McMaster University, Hamilton, ON Canada; 3 Department of Otolaryngology, Western University, London, ON Canada; 4 Faculty of Medicine, University of British Columbia, Vancouver, Canada

**Keywords:** COVID-19, Aerosol, Guideline, Aerosolization, Review

## Abstract

**Background:**

Aerosol generating medical procedures (AGMPs) present risks to health care workers (HCW) due to airborne transmission of pathogens. During the COVID-19 pandemic, it is essential for HCWs to recognize which procedures are potentially aerosolizing so that appropriate infection prevention precautions can be taken. The aim of this literature review was to identify potential AGMPs in Otolaryngology - Head and Neck Surgery and provide evidence-based recommendations.

**Methods:**

A literature search was performed on Medline, Embase and Cochrane Review databases up to April 3, 2020. All titles and abstracts of retrieved studies were evaluated and all studies mentioning potential AGMPs were included for formal review. Full text of included studies were assessed by two reviewers and the quality of the studies was evaluated. Ten categories of potential AGMPs were developed and recommendations were provided for each category.

**Results:**

Direct evidence indicates that CO2 laser ablation, the use of high-speed rotating devices, electrocautery and endotracheal suctioning are AGMPs. Indirect evidence indicates that tracheostomy should be considered as potential AGMPs. Nasal endoscopy and nasal packing/epistaxis management can result in droplet transmission, but it is unknown if these procedures also carry the risk of airborne transmission.

**Conclusions:**

During the COVID-19 pandemic, special care should be taken when CO2 lasers, electrocautery and high-speed rotating devices are used in potentially infected tissue. Tracheal procedures like tracheostomy and endotracheal suctioning can also result in airborne transmission via small virus containing aerosols.

## Background

In the era of globalization, infectious disease outbreaks have brought unprecedented challenges to the medical community. Coronavirus disease 2019 (COVID-19), the clinical condition caused by infection with severe acute respiratory syndrome coronavirus 2 (SARS-CoV-2), rapidly became the world’s 6th public health emergency of international concern (PHEIC) declared by the World Health Organization since 2009 [1]. The other PHEICs were the swine flu in 2009, polio in 2014, Ebola virus in 2014 and 2018–20, and Zika virus in 2016 [1]. SARS, small pox and wild type poliomyelitis are automatic PHEICs and do not require declaration from the WHO [2].

Although the main environmental route of transmission of SARS-CoV-2 is through droplets and formites/surfaces, there is a potential risk of virus spread in smaller aerosols during various medical procedures causing airborne transmission [3–6]. “Airborne transmission” refers to transmission of infection via small (< 5-10um) inspirable aerosols over extensive distances, whereas “droplet transmission” refers to transmission of infection by (larger) aerosols over short distances directly from the infected person to the susceptible person [7, 8].

Various procedures performed by Otolaryngologists to assess and/or treat patients may generate aerosols from areas of high viral shedding, such as the nasal and oral-pharyngeal cavity [6, 9]. Such aerosol generating medical procedures (AGMPs) can lead to close proximity transmission of aerosols, but also in the spread of small aerosols over extensive distances resulting in airborne transmission. According to colleagues in other countries such as China, Italy, and Iran, Otolaryngologists are among the highest risk group of contracting viruses while performing upper airway procedures without appropriate Personal Protective Equipment (PPE) [10]. As there is worldwide limited availability of PPE, it is essential to distinguish which procedures justify the use of high level, airborne precautions. The objective of this literature review is to identify potential AGMPs in Otolaryngology - Head and Neck Surgery (OHNS) and provide evidence-based recommendations.

## Methods

This manuscript followed the published methodology of developing an evidence-based review with recommendations by Rudmik et al. (2011) [11]. A literature search was performed on Medline, Embase and Cochrane Review Databases from inception to April 3, 2020. Given aerosol and droplet terminology has been used interchangeably in the literature, the search included both terms. A screening literature search was first performed using the search term (aerosol* or droplet*) and (procedure or treatment or surgery). The authors, J.H., A.H., C.L., J. P, YWQ, and P.Y. reviewed the articles for topics that pertained to the realm of the head and neck region. All abstracts were reviewed and the following inclusion criteria was applied: English articles, clinical or experimental studies involving procedures in the head and neck region. Studies were excluded if they were opinion papers, review papers, or if only the abstract was published (no manuscript available). This first review of papers led to the following procedures being identified: nasal endoscopy, nasal packing and treatment of epistaxis, endoscopic sinonasal and anterior skull base surgery, CO2 laser ablation, electrocautery, tracheotomy, endotracheal suctioning, oropharyngeal surgery, head and neck reconstruction surgery, dental procedures, mastoid surgery and nebulizer/atomizer. A second focused literature search was performed for each of the aforementioned procedures using the search term (aerosol* or droplet*) and the synonyms of the procedure (e.g. (aerosol or droplet) and (mastoidectomy or mastoid* or mastoid surgery)). The same inclusion and exclusion criteria were applied except this time procedures that were in the head and neck region but not performed by an Otolaryngologist were removed (example: irrigation wash in dental procedure). This was done in order to ensure no further articles were missed on the first search and to keep articles chosen were relevant to the audience of interest. Review papers were also cross referenced to ensure all studies were identified.

The included articles were categorized into various potential AGMP procedures. In this review an AGMP is defined as a medical procedure which has the potential to generate small (< 5-10um) aerosols that can travel greater than 2 m, and therefore an AGMP confers the potential for airborne transmission. In contrast, we defined droplet transmission as involving (larger) aerosols over short distances (< 2 m) directly from the infected person to the susceptible person via mechanisms such as coughing and sneezing. Each AGMP procedure category was assigned to a practicing Otolaryngologist Head & Neck Surgeon to review the evidence found in the articles, grade the evidence of the articles and develop recommendations for practice. The recommendations were created based on study design, the quality of research, directness of evidence and finally the balance between the potential harm of the procedure and the quality of evidence [12]. Since the potential harm of aerosolizing viable pathogens can have a large impact on the safety of HCWs, a strong recommendation can still be warranted despite low or very low confidence in effect estimates [12]. Direct and high quality evidence was defined as studies evaluating directly, or indirectly particles/aerosol concentrations in air samples. Indirect evidence could be obtained from experimental cadaver models, or retrospective epidemiological data. The manuscript then underwent an iterative review process in the following order: M. L, C.D., J.L., D.D.S., L. S and A.T.

## Results

**Fig. 1:**
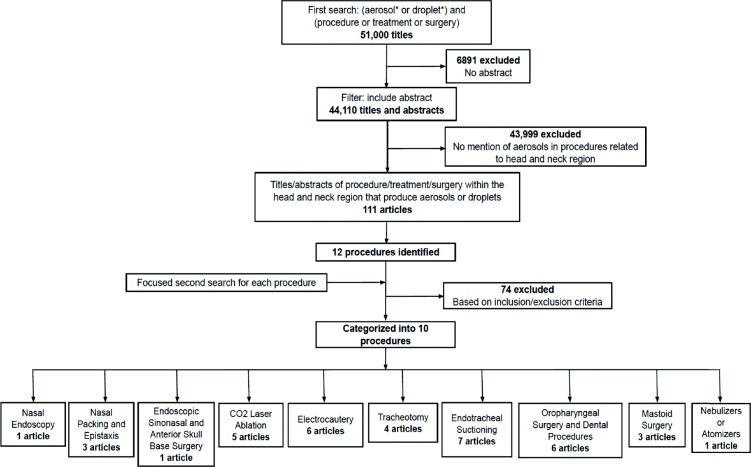
Evidence Based Review Search Strategy. OHNS = Otolaryngology – Head and Neck Surgery

The first literature search retrieved 44,110 articles (titles and abstracts), which were screened for potential eligibility. From this, 111 papers fit the inclusion/exclusion criteria, categorized into similar procedures and the second focused search was performed leading to 10 categorical procedures for review (oropharyngeal surgery, head and neck reconstruction surgery, dental procedures summarized under one heading for simplicity) (Fig. 1). The procedures and their evidence were then summarized below.

### Nasal Endoscopy

Only one study evaluating the aerosolization risk during nasal endoscopy was identified (Table 1). Workman et al. (2020) simulated potential aerosolization events using a cadaver with the nasal mucosa coated with fluorescein over a range of endoscopic procedures [13]. The potential aerosolization risk was quantified using a cadaveric model with fluorescein, a blue-light filter and digital image processing. The paper concludes nasal endoscopy did not generate aerosols; however, simulated coughing and sneezing using an atomization device did [13]. The tip of the atomizer was placed posterior to the internal valve, which may not accurately represent a true cough or sneeze. Nevertheless, activation of the atomizer device resulted in particle contamination up to 66 cm from the nare (produced droplet size: >30um), which by definition is droplet contamination. Both an intact surgical mask and a modified mask with a glove window were successful in eliminating all detectable spread of the particles [13].

**Table 1:** Characteristics of included studies on nasal endoscopy

Author, year	Study Design	Level of Evidence	Subjects (n)	Study Groups	Study outcomes	Conclusion	Directness of evidence
Workman et al. 2020 [13]	Experimental study	N/A	N/A	Cadavers	1. Visual inspection of number of fluorescein droplets generated by nasal endoscopy, endonasal surgery with and without microdebrider and high-speed drill. 2. Visual inspection of number of fluorescein droplets, generated by atomizer placed posterior to the internal valve (droplet size 30-100um) to simulate cough/sneeze.	1. Nasal endoscopy and cold non-powered endonasal procedures do not exhibit any features of AGMPs and has a lower risk of aerosol generation. 2. Droplet spread up to 66 cm from the nare, with peak density around 30 cm. (Modified) surgical masks were able to reduce the droplet spread.	Indirect

*AGMP* aerosol generating medical procedure

*Aggregated Evidence:* Grade D: one experimental study.

*Recommendation:*

Nasal endoscopy can cause coughing and sneezing of the patient, which may result in droplet transmission. It is unknown if this procedure can also lead to airborne transmission of small aerosols over extensive distances. Nasal endoscopy should be considered as a droplet forming procedure and as a potential AGMP.

*Strength of recommendation:* low.

### Nasal Packing and Treatment of Epistaxis

Three studies evaluated the risk of aerosol contamination during the treatment of epistaxis by visually examining blood contamination of the physician’s protective equipment [14–16]. All these studies confirmed that the treatment of epistaxis can cause transmission of blood aerosols within close proximity of the patient (Table 2). This is in line with the aerosol spread seen during coughing and sneezing, which generates aerosols in varying magnitudes and may contain pathogens [17, 18]. The above studies demonstrated that aerosol spread was significantly reduced if the patient wore a surgical mask during endoscopy or nasal packing [14, 15].

**Table 2:** Characteristics of included studies on nasal packing and treatment of epistaxis

Baig et al. 2015 [14]	RCT with high risk of bias	3	60	Adult patients presenting with epistaxis.	Number of blood spatters on surgical mouth mask and visor of physician (visual inspection)	Surgical face masks worn by patients covering their mouths decrease the risk of blood contamination.	Indirect
Hassan et al. 2003 [15]	Descriptive, cross sectional study	N/A	18	Adult patients presenting with epistaxis.	Number of blood spatters on surgical mouth mask, visor and gowns of physician (visual inspection)	Surgical face masks worn by patients covering their mouths decrease the risk of blood contamination.	Indirect
Wallace et al. 2002 [16]	Descriptive, cross sectional study	N/A	50	Adult patients presenting with epistaxis.	Number of blood spatters on protective glasses of physician (visual inspection)	Contamination of the protective glasses with blood occurred in 18% of cases.	Indirect

*RCT* randomized control trial

*AGMP*aerosol generating medical procedure

*Aggregated Evidence:* Grade C: one level 3 study, and two indirect, descriptive cross-sectional studies.

*Recommendation:* There is clinical evidence that treatment of epistaxis can cause coughing and sneezing of the patient, which may result in droplet transmission. It is unknown if these procedures can also lead to airborne transmission of smaller aerosols over extensive distances. Treatment of epistaxis and nasal packing should be considered as droplet forming procedures and as potential AGMPs.

*Strength of recommendation:* moderate.

### Endoscopic Sinonasal and Anterior Skull Base Surgery

Workman et al. (2020) investigated the aerosolization risk during endoscopic sinonasal procedures (Table 3). In their experimental design, no droplets were observed after performing cold non-powered endonasal procedures or with use of the microdebrider [13]. The authors hypothesized that the low aerosol spread with the microdebrider is due to the relatively low speed of rotation (in comparison to a drill) and the presence of a large-bore suction in the debrider [13]. The study does not explore the possibility of aerosol formation when the microdebrider suction is plugged but still rotating. The use of a high-speed powered drill did create high airflow velocities and was therefore considered aerosol generating and contamination was identified with both endonasal and external activation of the drill. The aerosol size produced from drilling was not stated. Closing or obstructing the nostrils during this procedure did not cease aerosol generation [13].

**Table 3:** Characteristics of included studies on endoscopic sinonasal and anterior skull base surgery

Author, year	Study Design	Level of Evidence	Subjects (n)	Study Groups	Study outcomes	Conclusion	Directness of evidence
Workman et al. 2020 [13]	Experimental study	N/A	N/A	Cadavers	Visual inspection of number of fluorescein droplets generated by nasal endoscopy, endonasal surgery with and without microdebrider and high-speed drill	High-speed drill can generate high airflow velocities and aerosolization. Nasal endoscopy and endonasal procedures, including the use of microdebrider do not exhibit any features of AGMPs and has a lower risk of aerosol generation	Indirect

*Aggregated Evidence:* Grade D: one experimental study.

*Recommendation:*

Based on limited evidence and clinical reasoning, powered instruments, which include the microdebrider and the drill, can result in droplet transmission and airborne transmission, and should be considered as droplet forming procedures and as AGMPs. Cold non-powered procedures are less likely to result in droplet or airborne transmission, as the patient is paralyzed during the procedure, and should be considered as potential droplet forming procedures, but not as AGMPs.

*Strength of recommendation:* low.

### CO_2_ Laser Ablation

Smoke samples have confirmed that laser ablation of tissue can generate aerosols [19]. There is consistent evidence revealing that HPV DNA can be present in the surgical smoke generated by CO2 lasers for the treatment of (laryngeal) papillomatosis and warts (Table 4) [20–23].

**Table 4:** Characteristics of included studies on CO2 laser ablation

Author, year	Study Design	Level of Evidence	Subjects (n)	Study Groups	Study outcomes	Conclusion	Directness of evidence
Genangeli, 2019 [18]	Experimental study	N/A	N/A	Different tissues, non-human	Mass spectrometry of air sample obtained using CO2 laser on different tissues	CO2 lasers can generate aerosols with detectable molecular profiles for all tissues tested	Direct
Kashima, 1991 [20]	Descriptive, cross sectional study	N/A	22	Patients with recurrent respiratory laryngeal papillomatosis	PCR of air samples for HPV DNA	1. 17/30 vapor samples were positive for HPV. 14 paired tissue and vapor samples revealed the same HPV type. 2. HPV-DNA in the vapor can be of concern to the operating team.	Direct
Garden, 1988 [21]	Descriptive, cross sectional study	N/A	7	Patients with plantar or mosaic verrucae	Electrophoresis and visualization of HPV DNA in air samples	1. Intact human papillomavirus DNA was present in the vapor for two of the seven patients. 2. Viral DNA can be released during the laser treatment for verrucae, even with clinically relevant laser parameter settings.	Direct
Sawchuk, 1989 [22]	Experimental study	N/A	8	Human plantar warts	Dot-blot analysis of HPV DNA in air samples	Five of eight laser-derived vapors were positive for HPV DNA.	Direct
Gloster, 1995 [23]	Case control study	4	31/6124	CO2 laser surgeons and patients with warts	Incidence of HPV lesions in CO2 laser surgeons	The overall incidence of acquired HPV warts, was not significantly different from the incidence of control patients. However, the incidence of nasopharyngeal warts was higher in CO2 laser surgeons (13%) compared to the control population (0.6%).	Indirect

*PCR* polymerase chain reaction

*HPV* Human papilloma virus

*DNA* deoxyribonucleic acid

*Aggregated Evidence:* Grade C: two direct, cross-sectional studies, one indirect level 4 study, and two experimental studies.

*Recommendations:* Consistent, direct evidence indicates that CO2 laser ablation of infected tissue can result in the spread of small, virus containing, aerosols. It is unclear if these aerosols can spread over longer distances, but given the small particle size generated by laser ablation, it is plausible. Laser ablation (CO2) should be considered as a droplet forming procedure and an AGMP.

*Strength of recommendation:* strong.

### Electrocautery

Three studies assessed particle concentrations during electrocauterization (Table 5) [24, 26, 27]. One cross-sectional study and two experimental studies investigated the potential of virus transmission by surgical smoke produced by electrocautery (Table 5) [22, 25, 28]. Electrocautery generates a high concentration of fine particles with diameters in the range of 10 nm to 1um [26]. There appears to be a direct positive relationship between the electrical current used during cauterization and particle concentration [24]. Higher current levels resulted in a significant increase in particle aerosolization. Ishihama et al. (2010) demonstrated the presence of aerosolized blood in the air vent filters of operating rooms after oropharyngeal, soft tissue cancer or reconstructive surgeries [27]. In 16 of 21 surgeries (76%) with identified aerosolized blood in the air filters, electrocauterization was used [27].

**Table 5:** Characteristics of included studies on electrocautery

Author, year	Study Design	Level of Evidence	Subjects (n)	Study Groups	Study outcomes	Conclusion	Directness of evidence
Carr, 2020 [24]	Descriptive, cross sectional study	N/A	36	Pediatric tonsillectomy patients	Airborne particle concentration in air sample during tonsillectomy.	Airborne particle concentration during tonsillectomy was over 9.5 times higher when electrocautery was set at 20 W compared to 12 W	Direct
Subbarayan, 2019 [25]	Descriptive, cross sectional study	N/A	6	Patients with resection of oropharyngeal cancer	PCR of air samples for HPV16 DNA	None of the electrocautery fumes sampled yielded detectable HPV16 DNA	Direct
Brüske-Hohlfeld, 2008 [26]	Descriptive, cross sectional study	N/A	6	Patients undergoing abdominal surgery	Airborne particle concentration in air samples	Electro-cauterization and argon plasma tissue coagulation induced the production of very high concentrations of particles in the diameter range of 10 nm to 1 μm.	Direct
Ishihama, 2010 [27]	Descriptive, cross sectional study	N/A	54	Patients undergoing head and neck surgeries.	Blood aerosols in OR air conduction filters	Surgical procedures using electrocautery can result in aerosolization of blood.	Direct
Sawchuk, 1989 [22]	Experimental study	N/A	7	Human plantar warts	Dot-blot analysis of HPV DNA in air samples	Four of seven electrocoagulation-derived vapors were positive for human papillomavirus DNA.	Indirect
Johnson, 1991 [28]	Experimental study	N/A	32 cell cultures	HIV-1 inoculated blood	Isolation of P^−24^ HIV-1 core antigen in cell cultures obtained from exposed to surgical smoked generated in the presence of HIV-1 inoculated blood	No HIV-1 was detected in cells exposed to surgical smoke	Indirect

*PCR* polymerase chain reaction

*HPV* Human papilloma virus

*DNA* deoxyribonucleic acid

*HIV* Human immunodeficiency virus

In contrast, Subbarayan et al. (2019) assessed the presence of viral DNA in electrocautery smoke produced during the resection of HPV16 positive oropharyngeal cancers [25]. PCR analysis of intraoperative smoke samples obtained from 6 different cases did not reveal HPV16 DNA [25]. This is in line with an experimental study assessing HIV-1 transmission via electrocautery smoke; the surgical smoke generated by cauterization of HIV-1 containing blood was collected and after 4 weeks of culturing no virus could be detected [28].

*Aggregated Evidence:* Grade C: four, direct cross-sectional studies, two experimental studies.

*Recommendations:* There is consistent, direct evidence indicating that electrocautery can result in small aerosols with potential spread over longer distances. It is uncertain if this can actually lead to clinically relevant transmission of viable pathogens. Electrocautery in tissue with potential high viral loads, i.e. aerodigestive tract, should be considered as a droplet forming procedure and as an AGMP.

*Strength of recommendation:* moderate.

### Tracheotomy

No studies performed air sample analyses during tracheotomies. In the 2009 retrospective cohort study by Chen et al. (2009), 6 of 17 HCWs performing tracheostomy developed SARS, conferring an odds ratio of 4.15 (univariate analysis 1.50 to 11.50, *p* < 0.01) [29]. However, in their multivariate analysis, tracheotomy was not a significant prognostic factor for the development of SARS [29]. It is unknown whether these infected HCWs were wearing full aerosol PPE, while performing the tracheotomies [29].

Three case series assessed the risk of SARS-CoV-1 infection during tracheotomies performed [30–32]. A total of 21 SARS-CoV-1 positive patients underwent tracheotomy whereby all HCWs used full aerosol PPE and no transmitted infections to HCWs were documented (Table 6) [30–32]. Tracheotomy, historically, has been considered a high risk aerosolizing procedure. This is in part due to the anesthesia literature illustrating high air flow within the trachea caused by endotracheal intubation, which may result aerosolization and thus an increased risk of virus transmission [29, 33–35].

**Table 6:** Characteristics of included studies on tracheotomies

Author, year	Study Design	Level of Evidence	Subjects (n)	Study Groups	Study outcomes	Conclusion	Directness of evidence
Chen, 2009 [29]	Retrospective cohort study	3	758	HCWs involved in care of SARS patients	Risk factors for SARS infection in HCWs, based on survey.	Univariate regression reveals increased OR for developing SARS: 4.15 (1.50–11.50), but this was not significant in their multivariate log regression analysis, which did not reveal an increased risk of performing tracheotomy.	Indirect
Wei, 2003 [30]	Cohort study, with high risk of bias	4	3	HCWs involved in SARS patients, requiring tracheotomies	SARS infection in HCWs, 3 tracheotomies	No medical personnel became infected after carrying out the procedure.	Indirect
Chee, 2004 [31]	Case control	4	124 HCWs	HCWs involved in care of SARS patients	SARS infection in HCWs. 41 surgical procedures, including 15 tracheotomies	No transmission of SARS was reported within the operating room	Indirect
Tien, 2005 [32]	Cohort study, with high risk of bias	4	3	HCWs involved in care of SARS patients	SARS infection in HCWs, 3 tracheotomies	Six months after the procedure, all staff involved in the tracheotomies remained healthy	Indirect

*HCWs* health care workers

*SARS* severe acute respiratory syndrome

*OR* odds ratio

*Aggregated Evidence:* Grade D: one level 3 study, and three level 4 studies with clinical reasoning.

*Recommendations:* There is only indirect evidence and expert opinion suggesting that tracheotomies are high risk of airborne transmission. Although there is paucity of evidence, tracheotomy should be considered as a droplet forming procedure and as an AGMP.

*Strength of recommendation:* strong.

### Endotracheal suctioning

Four studies revealed that air samples obtained during open endotracheal suctioning in mechanically ventilated patients result in higher concentrations of particulate matter and pathogens like bacteria, viruses and fungi (Table 7) [36–39]. Three retrospective studies, evaluating risk factors for SARS-CoV-1 transmission while providing patient care to SARS patients did not demonstrate that endotracheal suctioning was a significant risk factor for SARS infection [34, 40, 41]. However, in these series, HCWs were wearing adequate airborne protective equipment while providing patient care [34, 40, 41].

**Table 7:** Characteristics of included studies on endotracheal suctioning

Author, year	Study Design	Level of Evidence	Subjects (n)	Study Groups	Study outcomes	Conclusion	Directness of evidence
He, 2017 [36]	Environmental study	N/A	N/A	Air samples in PICU	Air samples from rooms in PICU were measured for particle concentration and mass.	Tracheal suction was a main indoor source for particle generation within PICU.	Direct
Chung, 2015 [37]	Environmental study	N/A	N/A	Air samples in respiratory centre	1. Air quality samples were taken over 1 year and measured for particle mass and concentration. 2. Agar plates were used to identify organisms found in the air	1. Open suctioning has been associated with raised levels of indoor air pollutants and bacteria. 2. The mean concentration of particulate matter increased significantly during open suctioning	Direct
Thompson, 2013 [38]	Descriptive, cross sectional study	N/A	39	Hospitalized patients with lower respiratory tract infections	RT-PCR of viral DNA in air samples	Respiratory/airway suctioning shows an increased risk of producing viral particles above baseline, but not statistically significant	Direct
Mousa, 2019 [39]	Descriptive, cross sectional study	N/A	10	Patients with Acinetobacter baumannii infection on ventilation	Cultures of air samples	Endotracheal suctioning increased the risk of air contamination	Direct
Loeb, 2004 [30]	Case control study	4	32/11	HCWs with/without SARS contact	SARS infection in HCWs	Suctioning after intubation was not associated with SARS infection rate.	Indirect
Raboud, 2010 [34]	Retrospective cohort study	3	624	HCWs with/without SARS contact	SARS infection in HCWs	Suctioning before and after intubation were not statistically significant risk factors for SARS transmission. However, a trend exists and may be associated with transmission.	Indirect
Teleman, 2004 [40]	Case control study	4	36/50	HCWs with/without SARS contact	SARS infection in HCWs	Suction of body fluids is not a procedure significantly associated with the development of SARS among HCWs	Indirect

*PICU* pediatric intensive care unit

*RT-PCR* real time polymerase chain reaction

*DNA* deoxyribonucleic acid

*HCWs* health care workers

*SARS* severe acute respiratory syndrome

*Aggregated Evidence:* Grade C: two direct cross-sectional studies, one level 3 and two level 4 studies and two environmental studies.

*Recommendations:* There is consistent evidence that endotracheal suctioning can result in the spread of small aerosols containing viable pathogens. Long distance spread of small aerosols remains possible under certain conditions. Endotracheal suctioning should be considered as a droplet forming procedure and as an AGMP (especially if the patient is mechanically ventilated).

*Strength of recommendation:* strong.

### Oropharyngeal Surgery and Dental Procedures

Three studies showed evidence of aerosol production during dental procedures and oropharyngeal surgeries within close proximity of the patient (within two meters) (Table 8) [27, 42, 45]. All of the studies indicated that the use of drills, saws and high pressure water sprays can increase the risk of aerosol formation [27, 42, 45]. One study by Ishihama et al. (2010) revealed evidence of aerosol transmission in a series of 33 operations [27]. Twenty-one surgeries revealed evidence of aerosol transmission of blood onto air vent filters [27]. In 95% of these surgeries (20/21), electrocautery or high-speed rotating instruments were used. In almost all (11/12) of the surgeries with no evidence of aerosolization, no electrocautery or high-speed rotating instruments were used [27]. Two other studies provided indirect evidence showing that the PPE of the HCW is frequently contaminated by blood aerosols (Table 8) [43, 44].

**Table 8:** Characteristics of included studies on oropharyngeal surgeries and dental procedures

Author, year	Study Design	Level of Evidence	Subjects (n)	Study Groups	Study outcomes	Conclusion	Directness of evidence
Ishihama, 2010 [27]	Descriptive, cross sectional study	N/A	54	Patients undergoing head and neck surgeries.	Blood-contaminated aerosols in OR air conduction filters	Surgical procedures using electrocautery and high-speed rotating instruments can result in aerosolization of blood.	Direct
Ishihama, 2009 [42]	Descriptive, cross sectional study	N/A	100	Patients undergoing third molar removal	Blood-contaminated aerosols in air samples	At 20 cm: 76% particles were blood contaminated, at 100 cm: 57%. Blood aerosols can be generated during oral surgery with high speed instruments	Direct
Ishihama, 2008 [43]	Descriptive, cross sectional study	N/A	25	Patients undergoing third molar removal	Blood-contamination of mask, visor and gown	Dental procedures with high-speed instruments exposed surgeons to possible blood-borne infections by splashing in nearly 90% of cases; blood splatters were confirmed in 84% of cases and 76% of visor masks	Indirect
Al-Eid, 2018 [44]	Descriptive, cross sectional study	N/A	30	Patients undergoing third molar removal	Blood-contamination of mask, visor and gown	Blood contamination was present for 100% of facemasks and gloves, 87% of protective eyewear, 73% of surgical gowns	Indirect
Hallier, 2010 [45]	Descriptive, cross sectional study	N/A	8	Patients undergoing dental procedures	Bacterial growth, caused by aerosol contamination, sampled 20 cm from dental chair.	Oral examination, tooth extraction, oral cavity preparation and ultrasonic scaling produce pathogen containing aerosols, which can form bacteria colonies	Direct
Perdelli, 2008 [46]	Experimental study	N/A	132	Patients undergoing dental procedures and maxillofacial surgery in the OR and experimental during autopsy	Hemoglobin concentration in air samples from dental cubicles, MFS operating room and autopsy room	Hemoglobin concentrations in air samples were highest in dental operations and lowest during autopsy procedures	Indirect

*OR* operative room

*MFS* maxillofacial surgery

*Aggregated Evidence:* Grade C: three direct, and two indirect cross-sectional studies, and one experimental study.

*Recommendations:* Consistent and direct evidence indicates the risk of small aerosol formation when high-speed rotating instruments are used in the oral cavity. One study also suggests that these aerosols can become airborne. The use of electrocautery and high-speed rotating instruments (powered instruments) within the oral cavity and pharynx should be considered as droplet forming procedures and as AGMPs.

*Strength of recommendation:* strong.

### Mastoid Surgery

The aerosolized bone dust and irrigation fluid produced during drilling of the mastoid bone can become a potential risk for transmission of disease. Various viruses, including coronaviruses, have been documented in the middle ear mucosa during active infections [47, 48]. There was significant heterogeneity among the three studies and only one study was deemed clinically relevant and of adequate quality [49]. The three studies were found describing aerosolization of bone dust and contaminated irrigation fluid (Table 9) [49–51].

**Table 9:** Characteristics of included studies on mastoid surgery

Author, year	Study Design	Level of Evidence	Subjects (n)	Study Groups	Study outcomes	Conclusion	Directness of evidence
Norris, 2011 [49]	Experimental study	N/A	3 Temporal bones	Respiratory airway, mannequin model	Concentration of particular matter in three conditions: without a mask, with a surgical mask, with an N95 respirator	The average concentration of bone dust particles with standard surgical masks was 1.66 mg/m^3^, compared with undetectable (< 0.81 mg/m^3^) with the use of an N95 respirator.	Indirect
Hilal, 2005 [50]	Experimental study	N/A	6 temporal bones		1.Penetration of bone dust and particulates onto the corneal surface of fish eyes placed 50 cm and 1 m away 2. bone dust scattering radius	All corneas examined (up to 1 m away) had numerous bone particles penetrating the corneal surface. In vivo, bone dust scatters 106 cm in all directions.	Indirect
Lannigan, 1989 [51]	Experimental study	N/A	Unknown		Maximum radius of black fluid spray of various drill speeds	Maximum radius of spray was 41 cm with a drill at 40,000 RPM.	Indirect

*RPM* revolutions per minute

*Aggregated Evidence:* Grade D: three experimental studies.

*Recommendations:* Indirect evidence from studies with varying quality indicate that drilling of the mastoid generates small aerosols. Mastoidectomy should be considered as a droplet forming procedure and an AGMP.

*Strength of recommendation:* moderate.

### Nasal Nebulizer/Atomizers

One experimental study was identified, which revealed that in 9 of the 15 subjects, bacterial contamination of the tip of Venturi atomizers was demonstrated (Table 10) [52].

**Table 10:** Characteristics of included studies on nebulizers/atomizers

Author, year	Study Design	Level of Evidence	Subjects (n)	Study Groups	Study outcomes	Conclusion	Directness of evidence
Tseng, 2014 [52]	Experimental study	N/A	15	Healthy subjects	Bacterial contamination of atomizer nozzle tip (bacterial cultures).	1. 18 out of 30 samples (60%) were positive for bacterial growth at the atomizer tip. 2. During the spray process, aerosols were noted traveling backwards through the reversed jet flow and attaching to the nozzle tip, contaminating the tip.	Indirect

*Aggregated evidence:* Grade D: one experimental study.

*Recommendations:* One experimental study suggests that the nozzle tips of powered atomizers can get contaminated but no evidence that the action of the atomizer generates aerosols from the patient. The use of atomizers/nebulizers should be considered droplet forming procedures given the risk of coughing and sneezing but not an AGMP.

*Strength of recommendation:* low.

## Discussion

Surgical procedures using (CO2) laser vaporization, electrocautery and/or high-speed powered rotating instruments, like microdebriders, drills and saws, can result in airborne transmission of aerosols and should therefore be considered AGMPs. In addition, endotracheal procedures like endotracheal suctioning and tracheotomies should also be considered AGMPs as high tracheal airflow can result in airborne transmission of small aerosols. Nasal endoscopy, epistaxis management and in-office sinonasal procedures can induce sneezing and coughing of the patient. Although sneezing and coughing is considered to result in mainly droplet transmission, HCWs should be aware that the resulting clouds also contain small, inspirable aerosols, which can impose a risk, when they are working in close proximity to the patient [4, 7, 8].

For the protection of HCWs during this COVID-19 pandemic, it is not only essential to recognize which procedures are aerosolizing, but physicians should also be aware of the distribution of SARS-CoV-2 throughout the body. The highest viral loads of SARS-CoV-2 have been found in the upper and lower airways, but the virus has also been identified in feces [53]. Viral RNA has even been found in the blood of both symptomatic and asymptomatic COVID-19 patients, and as such, inhaled aerosol of blood may potentially transmit infection [54, 55]. SARS-CoV-2 appears to be quite sensitive to temperature, being largely inactivated at temperatures above 70 °C [56]. This is important given the high temperature of electrocautery may result in nonviable virus in the plume. This matter is of great importance, not just to our specialty, but to all surgeons operating in this era.

For Otolaryngologists performing aerosol generating procedures, guidelines on PPE use have been suggested in SARS-CoV-2 positive or suspected patients [57, 58]. Although coughing and sneezing mainly results in the emission of larger droplets, the risk of inhaling potentially smaller SARS-CoV-2 infected aerosols should not be neglected, especially if the HCW is in close proximity. For in-office endonasal procedures and nasopharyngoscopy, in the vast majority of patients, standard level 2 airborne PPE, including N95 masks are recommended. We further recommend keeping as much distance from the patient by using video endoscopy instead of the eye-piece. On the other hand, aerosolizing procedures in SARS-CoV-2 positive or suspected patients warrant extreme airborne precautions and level 3 PPE is recommended by our working group, which include either powered air purifying respirators (PAPR) or body/face/eye protection with N99/FFP3 respirators (99% filtration rate, or 95% if not available) [57, 58]. In addition, it is recommended that AGMPs are performed in negative pressure rooms to minimize the risk of spread of contaminated aerosols.

One of the major limitations of this review is the fact that most recommendations can only be based on evidence from small, descriptive case-series, experimental studies or indirect retrospective cohort studies. Even if direct evidence was available, the clinical applicability of the various study results can be questioned during the current COVID-19 pandemic. Virus kinetics can differ significantly and this not only includes the potential to survive in aerosols, but also its sensitivity to heat and shear stress during drilling. Extrapolating evidence obtained from studies investigating the potential of airborne transmission and infection of HPV or influenza virus might not hold true for SARS-CoV-2.

Given the limitations of the available research and knowledge surrounding this topic, we recommend HCWs err on the side of caution. As the risks of potential infection with SARS-CoV-2 are significant, a careful balance between the potential harms of the procedure and quality of the available evidence was considered in these recommendations. Therefore, a strong recommendation that a procedure is an AGMP can be provided despite the paucity of high quality and direct evidence. For example, regarding endoscopic use of microdebriders, due to high-speed rotation of the blade, the authors recommend the microdebrider be considered an AGMP as clinical experience intraoperatively conveys frequent microdebrider suction port plugging. To minimize the risk, we suggest the placement of a suction in the nose or suction catheter in the nasopharynx either in the contralateral nostril or via the oropharynx. The use of the suction near the surgical field is also recommended when using electrocautery or the CO2 laser. Furthermore, given the evidence that endotracheal suctioning is an AGMP, there is a similar theoretical risk of fenestrated suction use in the oral, ear and nasal cavity, for which we suggest the use of level 2 PPE precautions.

These recommendations have been developed by reaching consensus between the authors during the COVID-19 pandemic. A summary of included procedures and recommendations are provided in Table 11. Several gaps in knowledge exist regarding OHNS procedures and the nature of aerosol generation, and further research is needed to provide higher quality evidence based recommendations. As our understanding of COVID-19 evolves and literature grows regarding aerosol generation of various procedures, the above recommendations will need to be revised. Further research is required in the field of OHNS to help our specialty get through this pandemic and better equip us for the next.

**Table 11:** Summary of included procedures and recommendations

Procedure	Droplet (Y/N/Potential)	AGMP (Y/N/Potential)	Aggregated Evidence	Strength of Recommendation
Nasal Endoscopy	Y	Potential	D	Low
Nasal Packing and Treatment of Epistaxis	Y	Potential	C	Moderate
Endoscopic Sinonasal and Anterior Skull Base Surgery	1. Powered instruments: Y 2. Cold non-powered procedures: Y	1. Powered instruments: Y 2. Cold non-powered procedures: N	D	Low
CO2 Laser Ablation	Y	Y	C	Strong
Electrocautery	Y	Y	C	Moderate
Tracheotomy	Y	Y	D	Strong
Endotracheal Suctioning	Y	Y	C	Strong
Oropharyngeal Surgery and Dental Procedures	Y	Y	C	Strong
Mastoid Surgery	Y	Y	D	Moderate
Nasal Nebulizer/Atomizers	Y	N	D	Low

*AGMP* aerosol generating medical procedure

*Y* yes

*N* no

## Conclusion

During the COVID-19 pandemic, tracheotomy, endotracheal suctioning, the use of high-speed rotating devices, CO2 lasers and electrocautery on aerodigestive tissue should be considered AGMPs.
